# Loss of a child and the risk of atrial fibrillation: a Danish population-based prospective cohort study

**DOI:** 10.1136/jech-2022-219695

**Published:** 2023-03-01

**Authors:** Dang Wei, Imre Janszky, Jiong Li, Krisztina D László

**Affiliations:** 1 Institute of Environmental Medicine, Karolinska Institutet, Stockholm, Sweden; 2 Department of Global Public Health, Karolinska Institutet, Stockholm, Sweden; 3 Department of Public Health and Nursing, Norwegian University of Science and Technology, Trondheim, Norway; 4 Department of Clinical Medicine - Department of Clinical Epidemiology, Aarhus University, Aarhus, Denmark; 5 Department of Public Health and Caring Sciences, Uppsala University, Uppsala, Sweden

**Keywords:** STRESS, CARDIOVASCULAR DISEASES, EPIDEMIOLOGY

## Abstract

**Background:**

Several studies suggest that bereavement is associated with increased risks of ischaemic heart disease, heart failure, stroke and cardiovascular mortality. Knowledge regarding the link between bereavement and the risk of atrial fibrillation (AF) is limited. We investigated whether the death of a child, one of the most severe forms of bereavement, is associated with AF.

**Methods:**

We conducted a population-based cohort study involving parents of live-born children during 1973–2016 from the Danish Medical Birth Register (n=2 804 244). Information on children’s death, parental AF and sociodemographic and other health-related characteristics was obtained by individual-level linkage between several Danish population-based registers. We analysed the association between loss of a child and AF using Poisson regression.

**Results:**

During the up to 39 years follow-up, 64 216 (2.3%) parents lost a child and 74 705 (2.7%) had an AF. Bereaved parents had a higher risk of AF than the non-bereaved; the corresponding incidence rate ratio (IRR) and 95% CI were 1.12 (1.08 to 1.17). The association was present both when the child died of cardiovascular diseases (IRR (95% CI): 1.42 (1.20 to 1.69)), and of other causes (IRR (95% CI): 1.11 (1.06 to 1.16)), tended to be U-shaped according to the deceased child’s age at loss, but did not differ substantially according to the number of remaining live children at loss, the number of deceased children or the time since the loss.

**Conclusions:**

The death of a child was associated with a modestly increased risk of AF. Bereaved parents may benefit from increased support from family members and health professionals.

**WHAT IS ALREADY KNOWN ON THIS TOPIC**

- Increasing evidence suggests that bereaved individuals are at increased risks of ischaemic heart disease, heart failure and stroke. Knowledge regarding the link between bereavement and the risk of atrial fibrillation remains limited.

**WHAT THIS STUDY ADDS**

- In this large population-based cohort study involving more than 2.8 million parents from Denmark, we found that parents who lost a child had a higher risk of atrial fibrillation than non-bereaved parents. The association was observed irrespective of the child’s cause of death, though it was stronger if the child died of cardiovascular diseases than of other causes. The association tended to be U-shaped according to the deceased child’s age at loss, but did not differ substantially according to the number of remaining live children at loss, the number of deceased children or the time since the loss.

**HOW THIS STUDY MIGHT AFFECT RESEARCH, PRACTICE OR POLICY**

- Further studies are needed to investigate whether our findings may be generalised to other socioeconomic and cultural contexts and to understand the mechanisms underlying the observed association. Bereaved parents may benefit from increased support from family members and health professionals.

## Background

Increasing evidence suggests that bereavement, a severely stressful life event that most individuals will experience at least once in life, is associated with increased risks of cardiovascular diseases (CVDs), including ischaemic heart disease, heart failure and stroke.^1–6^ Knowledge regarding the link between bereavement and the risk of atrial fibrillation (AF), the most common cardiac arrhythmia, is limited as only two studies analysed this association.^7 8^ Graff *et al* found in a Danish register-based study a transiently increased risk of AF in the year after the death of a partner,^8^ while in a Swedish study, we reported that parents who lost a child had an increased risk of AF both on the short and on the long term.^7^ Our findings that the association was present not only in case of the child’s cardiovascular deaths, but also in case of deaths less likely to be affected by familial cardiovascular risk factors, and that the risk of AF was highest in the week immediately after the loss, are suggestive of stress as a potential explanation.^7^ Acute stress and the activation of the hypothalamic–pituitary–adrenocortical axis may trigger paroxysmal AF episodes,^9^ while chronic stress may lead to depression,^10^ anxiety, post-traumatic stress disorder,^11 12^ alcohol or drug abuse,^13^ and to adverse changes in the cardiovascular, haemostatic, metabolic and immune systems,^14–16^ which may promote structural and electrical changes in the heart and the development of AF.^7 17 18^

In this large population-based study in Denmark, we analysed whether the death of a child is linked with an increased risk of AF and whether the association differs by the characteristics of the loss and the time since the loss.

## Methods

### Study population and design

**Figure 1:**
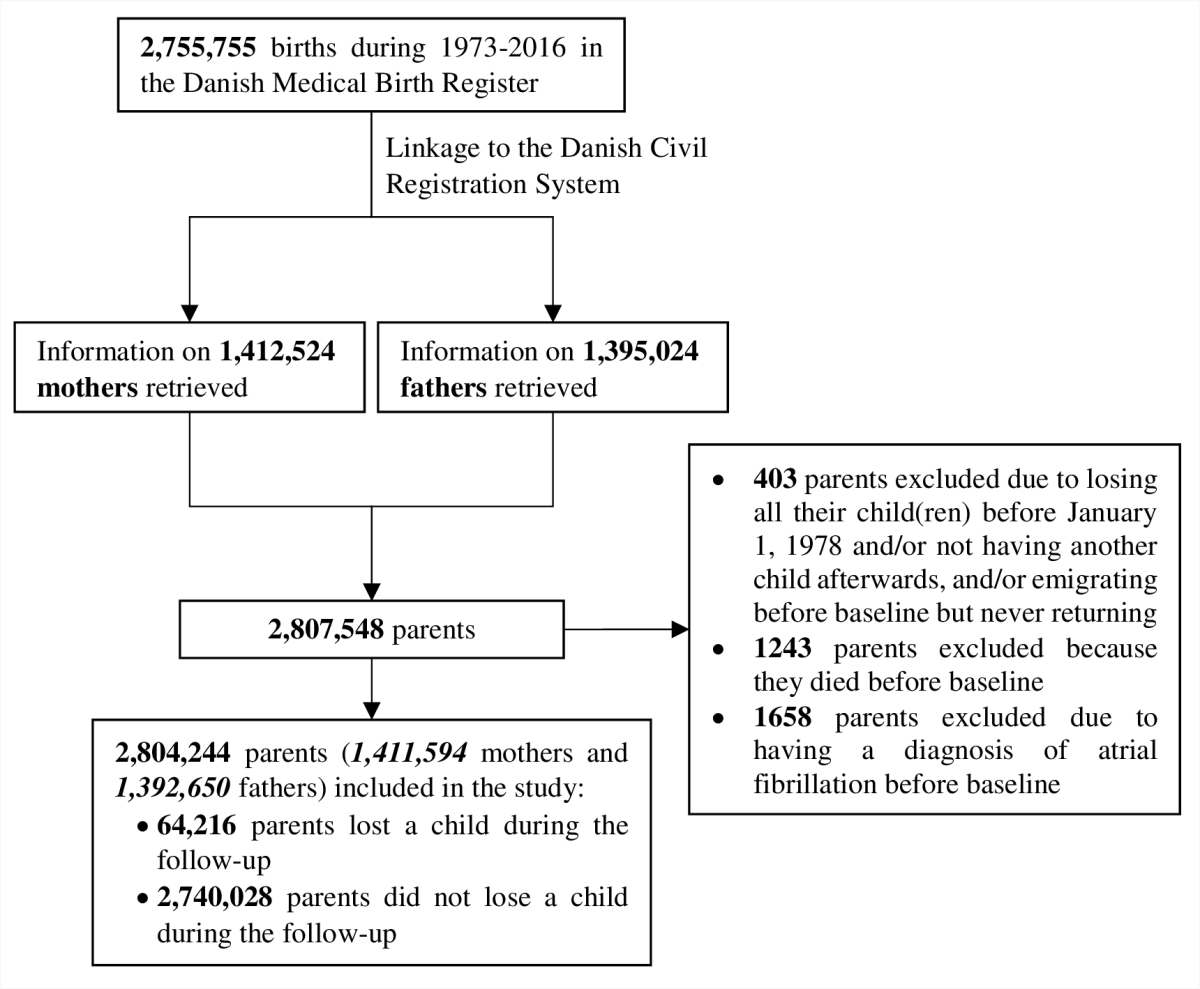
Flow chart of the study participants.

We performed a population-based cohort study by merging data from several Danish national registers. Linkage between registers was possible through the social identification number, unique for each resident. Our study population consisted of parents of 2 755 755 live-born children during 1973–2016, recorded in the Danish Medical Birth Register (MBR) (figure 1). We identified in the MBR the mothers of virtually all children in the MBR, whereas to identify fathers we combined information from the MBR and the Danish Civil Registration System (CRS); we identified in this way the fathers of 98.9% of the children from the MBR.^4^ Using the CRS, we also identified the studied parents’ children that were not recorded in the MBR, either because they were born before the MBR was established or were born outside of Denmark.^2 4 6^ Since the Danish National Hospital Register (NHR), which was established in 1977 and from which we identified the diagnoses of AF, became nationwide in 1978, we defined our study period as 1978–2016. Parents were eligible for this study, if they were alive and resided in Denmark at baseline, and if they had at least one live child any time during the study period (n=2,805,902). There were several ways to enter the study. Parents with one or more live children on 1 January 1978, entered the cohort on this date; parents who gave birth later entered the cohort on the date of birth of the first child. If parents immigrated to Denmark with their child(ren) after 1978 and later had at least one child recorded in the MBR, they entered the cohort on the date of the registered immigration. We excluded parents who had an AF diagnosis in the NHR before the study entry, resulting in 2 804 244 parents (1 411 594 mothers and 1 392 650 fathers) being included in the analyses (figure 1).

### Exposure

Study participants were considered exposed if they lost a child during the follow-up. We obtained data on children’s date and cause of death from the CRS. We treated exposure as a time-varying exposure, that is, study participants who lost a child were considered unexposed until the child’s date of death and exposed afterwards. Study participants who did not lose a child were considered unexposed throughout the whole study period. If study participants lost more than one child during the follow-up, we considered the first loss in the main analyses.

### Outcome

We identified incident AF by searching the primary and the secondary diagnoses in both inpatient and outpatient care in the NHR for the following International Classification of Disease (ICD) codes: ICD-8: 427.93, 427.94 and ICD-10: I48 (online supplemental table 1). The NHR contains information on all diagnoses given in inpatient care since 1977 and in specialised outpatient care since 1995. A validation study has shown that the positive predictive value of the AF diagnosis in the NHR is high (92.6%).^19^ We followed study participants from study entry until the first AF, emigration, death or 31 December 2016, whichever came first.

10.1136/jech-2022-219695.supp1 Supplementary data

### Covariates

We retrieved information on study participants’ sex, age, country of birth, marital status, education and income from several Danish nationwide registers, as described in online supplemental table 2. We replaced missing data on marital status, education and income at baseline with the closest available information from the 5 years prior to study entry. As the Danish Integrated Database for Longitudinal Labor Market Research was established in 1980, we defined education and income based on information from 1980 for those who entered the cohort before 1981.^2 4 6^ To consider inflation over time we categorised income into tertiles within each 10-year interval.^4^

We obtained data on family history of CVD by identifying study participants’ parents and siblings in the CRS and retrieving information on their history of CVD from the NHR and the CRS. The CRS, established in 1968, contains links to parents for index persons born in 1960 or later and links to children for index persons born in 1935 or later.^20^ We retrieved data on study participants’ history of CVD from the NHR and of psychiatric disorders from the NHR and the Danish Central Psychiatric Register. We retrieved data on maternal hypertension and diabetes before childbirth from the NHR, given that only women of reproductive age are screened for these medical conditions, when pregnant. Data on smoking were available only for women, in early pregnancy, in the MBR.^2 4 6^ Information on the source of each variable and the time periods with available data is described in online supplemental table 2.

### Statistical analyses

We analysed the association between the death of a child and the risk of AF by estimating incidence rate ratios (IRRs) and 95% CI using Poisson regression. In our main multivariable models, we adjusted for age and calendar year at follow-up (as time-varying variables), sex, country of birth, highest educational attainment, history of psychiatric disorders and CVD at baseline. To study whether the risk of AF differed by the child’s cause of death, we classified bereaved parents in two groups, that is, death due to CVD or other causes, using the ICD codes shown in online supplemental table 1. We also investigated whether the association between the loss of a child and the risk of AF differed by the age of the deceased child at loss (categorised as ≤1, 2–12, 13–18, 19–29 and >29 years), the number of remaining live children at the time of loss (categorised as 0, 1–2 and ≥3) and the number of deceased children during the follow-up (categorised as 1 and >1). Even in the analysis concerning the number of deceased children during the follow-up, we treated exposure as time-varying. Accordingly, parents who lost two or more children contributed person-time (1) to the unexposed group from study entry until the date of the first loss, (2) to the exposed group with one loss from the date of the first to that of the second loss and (3) to the exposed group with two or more losses afterwards. Study participants who lost a child were considered unexposed until the child’s date of death and exposed to one loss afterwards. Study participants who did not lose a child were considered unexposed throughout the whole study period. We compared effect sizes corresponding to the different exposure categories using the contrast statement in Poisson regression model in SAS.

To analyse whether the strength of the association differed according to the time since the loss, we estimated separate IRRs and 95% CIs for the following periods: 0–3 months, 4–12 months, 2–5 years, 6–10 years and >10 years after the loss.

Due to missing information on a large number of study participants on marital status, income and family history of CVD, we adjusted for these variables in sensitivity analyses among those with available data. Similarly, as women were screened for maternal hypertension and diabetes during pregnancy and information on maternal smoking was recorded in early pregnancy, we adjusted for these variables in the subcohort of women with data available on these variables. We performed stratified analyses and tests of interaction with sex, age, education and the year of study entry; study entry was classified according to the year when the NHR became nationwide (before 1978 vs 1978 or later) and when the information on specialised outpatient care was included in the NHR (before 1995 vs 1995 or later).

We performed all analyses in SAS 9.4.

## Results

A total of 64 216 (2.3%) study participants lost a child during the study period. Compared with their unexposed counterparts, bereaved individuals were more likely to be women, born in Denmark, enter the cohort in earlier years of the study period, married or in registered partnership, to have lower education and income, less likely to have a history of CVD and psychiatric disorders as well as a family history of CVD (table 1).

**Table 1:** Characteristics of study participants according to exposure to death of a child

Variables	Exposure to the loss of a child	
	Unexposed (n=2 740 028)	Exposed (n=64 216)
	N (%)	N (%)
All cohort		
Age at study entry/mean (SD), years	29.0 (5.4)	28.5 (5.8)
Sex		
Men	1 362 908 (49.7)	29 742 (46.3)
Women	1 377 120 (50.3)	34 474 (53.7)
Country of birth		
Denmark	2 428 681 (88.6)	58 462 (91.0)
Other countries	311 347 (11.4)	5754 (9.0)
Year of entry in the study		
Before 1980	698 879 (25.5)	31 510 (49.1)
1980–1989	502 426 (18.3)	15 724 (24.5)
1990–1999	596 641 (21.8)	9992 (15.6)
2000–2009	565 652 (20.6)	5140 (8.0)
After 2009	376 430 (13.7)	1850 (2.9)
Marital status at baseline*		
Married or in registered partnership	1 402 329 (51.2)	34 977 (54.5)
Single, widowed or divorced	451 847 (16.5)	7073 (11.0)
Missing	885 852 (32.3)	22 166 (34.5)
Highest education at baseline (in years)†		
0–9	711 221 (26.0)	26 339 (41.0)
10–14	1 276 509 (46.6)	25 956 (40.4)
≥15	582 730 (21.3)	8314 (12.9)
Missing	169 568 (6.2)	3607 (5.6)
Household income at baseline‡		
Low tertile	618 113 (22.6)	11 175 (17.4)
Middle tertile	619 603 (22.6)	9691 (15.1)
High tertile	620 919 (22.7)	8098 (12.6)
Missing	881 393 (32.2)	35 252 (54.9)
History of CVD at baseline		
No	2 679 130 (97.8)	63 338 (98.6)
Yes	60 898 (2.2)	878 (1.4)
History of psychiatric disorders at baseline		
No	2 663 390 (97.8)	63 151 (98.3)
Yes	76 638 (2.8)	1065 (1.7)
Parents’ history of CVD§		
No	1 227 013 (44.8)	25 470 (39.7)
Yes	671 916 (24.5)	8851 (13.8)
Missing	841 099 (30.7)	29 895 (46.6)
Sibling’s history of CVD§		
No	1 794 567 (65.5)	33 222 (51.7)
Yes	104 362 (3.8)	1099 (1.7)
Missing	841 099 (30.7)	29 895 (46.6)
Maternal subcohort (n=1 411 594)		
Maternal hypertension before childbirth		
No	1 350 442 (98.1)	34 027 (98.7)
Yes	26 678 (1.9)	447 (1.3)
Maternal diabetes before childbirth		
No	1 368 559 (99.4)	34 343 (99.6)
Yes	8561 (0.6)	131 (0.4)
Maternal smoking in early pregnancy¶		
No	551 778 (40.1)	5118 (14.8)
Yes	135 176 (9.8)	1918 (5.6)
Missing	690 166 (50.1)	27 438 (79.6)

*The information on marital status was available since 1972.

†The information on education was available since 1980.

‡The information on income was available since 1980.

§The information on the linkage to parents and siblings was available since 1970, while on CVDs from 1977.

¶The reason for the high missing rate on maternal smoking is that information on this variable started to be registered in 1991 and even during subsequent years its coverage was not complete and varied over the years. A total of 885 643 women had at least one child in 1991 or later.

CVD, cardiovascular diseases; SD, standard deviation.

**Figure 2:**
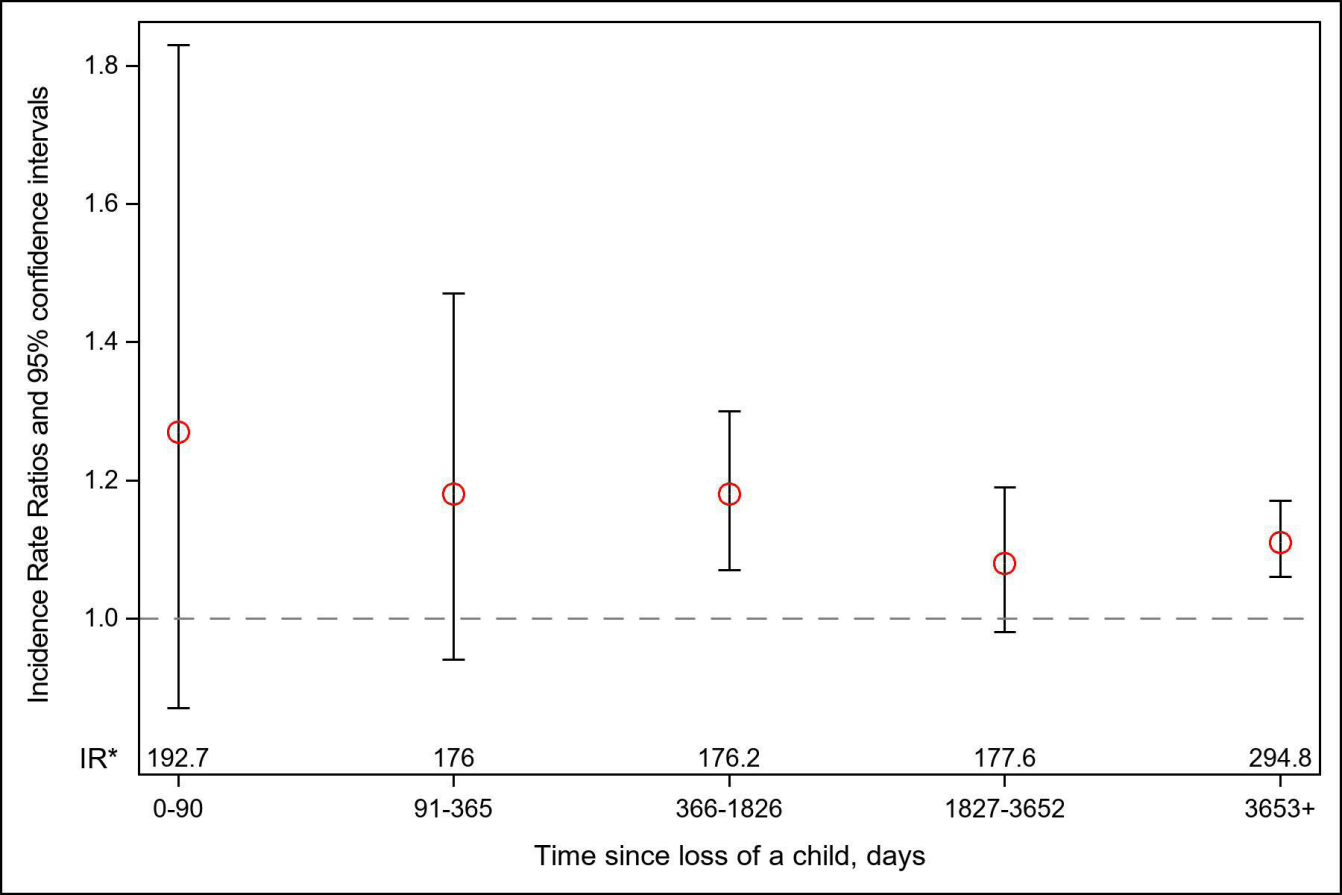
Adjusted incidence rate ratios and 95% CIs for atrial fibrillation according to time since the death of a child. We adjusted for sex, age at follow-up, calendar year at follow-up, country of birth, highest educational attainment, history of psychiatric disorders and of cardiovascular diseases. The incidence rate of atrial fibrillation (per 10^5^ person-years) for the exposed group. *IR, incidence rate.

Altogether 74 705 (2.7%) study participants had an AF during the up to 39 years follow-up; the incidence rates were 234.4 in the exposed and 121.2 per 10^5^ person-years in the unexposed groups. The death of a child was associated with a modestly increased risk of AF (IRR (95% CI): 1.12 (1.08 to 1.17)). The association was stronger in case the child died due to CVD (IRR (95% CI): 1.42 (1.20 to 1.69)) than due to other causes (IRR (95% CI): 1.11 (1.06 to 1.16)). We observed a trend toward a U-shaped association when we categorised exposure according to the deceased child’s age at loss (table 2). The association did not differ substantially according to the number of remaining live children at loss or the number of deceased children (table 2) or the time since loss (figure 2).

**Table 2:** Adjusted IRRs and 95% CIs for atrial fibrillation according to characteristics of the exposure to the death of a child

Exposure	No of events	Rate/10^5^ person-year	Age-adjusted IRR (95% CI)	Multivariable IRR (95% CI)*	P value†
Unexposed	72 232	121.2	1.00	1.00	–
All deaths	2473	236.4	1.10 (1.07 to 1.15)	1.12 (1.08 to 1.17)	–
Cause of death of the deceased					
Death due to CVD	139	569.6	1.34 (1.13 to 1.58)	1.42 (1.20 to 1.69)	Reference
Death due to other causes	2334	228.5	1.09 (1.05 to 1.14)	1.11 (1.06 to 1.16)	<0.0001
Age of the deceased child at loss (in years)					
≤1	624	122.1	1.27 (1.18 to 1.38)	1.17 (1.08 to 1.27)	0.0118
2–12	337	169.7	1.10 (0.98 to 1.22)	1.07 (0.96 to 1.19)	0.2041
13–18	193	244.0	0.94 (0.82 to 1.09)	0.95 (0.82 to 1.10)	Reference
19–29	655	370.1	1.00 (0.93 to 1.08)	1.04 (0.96 to 1.13)	0.2573
>29	664	826.7	1.14 (1.05 to 1.23)	1.26 (1.17 to 1.36)	0.0005
No of remaining live children at loss					
0	375	133.2	1.25 (1.13 to 1.39)	1.13 (1.02 to 1.26)	0.5530
1–2	1578	248.2	1.09 (1.03 to 1.14)	1.09 (1.04 to 1.15)	Reference
≥3	520	403.7	1.07 (0.98 to 1.16)	1.21 (1.11 to 1.32)	0.0508
No of losses during follow-up‡					
1	2353	228.6	1.10 (1.06 to 1.15)	1.12 (1.07 to 1.17)	Reference
≥2	95	240.7	1.10 (0.90 to 1.34)	1.12 (0.91 to 1.38)	0.9956

*Adjusted for sex, age at follow-up, calendar year at follow-up, country of birth, highest educational attainment, history of psychiatric disorders and CVDs.

†We tested the differences in the strength of the associations between the different exposure categories and atrial fibrillation risk using the contrast statement in Poisson regression model in SAS. We set as reference group the group who lost a child due to CVDs in the analysis with cause of death, the group who lost a child aged 13–18 years in the analysis with age of the deceased child at loss, the group with 1–2 children left in the analysis with number of remaining live children at loss and the group who lost one child during follow-up in the analysis with the number of losses during follow-up.

‡The analyses were performed among those who had not lost any children before baseline.

CI, confidence interval; CVD, cardiovascular diseases; IRR, incidence rate ratio.

We did not find substantial differences in the association between bereavement and AF when we stratified our main analyses by sex, age, education or study entry (online supplemental table 3). The association between the death of a child and AF did not change (1) after adjusting for marital status, income, family history of CVD, maternal hypertension and diabetes before childbirth or maternal smoking in early pregnancy, nor (2) after excluding those who lost a child before study entry (online supplemental table 4).

## Discussion

In this large population-based cohort study, we found that the death of a child was associated with an increased risk of AF. The association was present not only if the child died of CVD but also in case of losses due to other causes and tended to be U-shaped according to the child’s age at loss; it did not differ according to the number of remaining live children at the time of the loss, the number of losses during the study period, or according to the time since the loss.

Findings from this study corroborate the results of our earlier investigation conducted in a Swedish population,^7^ reporting a 15% higher risk of AF in parents who lost a child than in their unexposed counterparts and that the association was present not only when the child died due to CVD, but also in case of deaths due to other causes. The association between the child’s non-cardiovascular death and AF was less likely to be prone to confounding by genetic and environmental cardiovascular risk factors shared by family members than that corresponding to losses due to CVD, supporting in both studies a stress-related mechanism.^7^ Our findings were also in line with those of previous studies suggesting that the death of a partner is associated with an increased AF risk^8^ and of studies showing that the death of a child was associated with increased risks of ischaemic heart disease, heart failure and stroke.^2–4 6^ Further, our findings corroborate the results of several studies regarding the link between less severe psychological stressors than bereavement, including job strain, perceived stress, and post-traumatic stress disorder, and an increased AF risk.^21^

The death of the only child may be particularly devastating as it involves the loss of the parental role, while having more than two children after the loss may be challenging as the parents need to combine their own grief work with caring for several children and supporting them in their grief.^2 4^ In line with this hypothesis, we reported earlier that bereaved parents with more than two children left at the time of the loss had higher risks of ischaemic heart disease, stroke, AF and heart failure than those with one or two remaining live children.^2 4 6 7^ When we categorised exposure according to the number of remaining children at the time of loss, we observed in this study a trend toward a similar U-shaped association, but due to the smaller sample size than in the previous studies the CIs corresponding to these exposure subcategories overlapped. Similarly, based on our earlier studies,^2 4^ we hypothesised that the longer time spent together and thus the stronger bond with the older than with the younger children may result in a higher risk of AF after loss of older than younger children. Partly in line with this hypothesis, we observed the strongest association in case of loss of a child older than 29 years. Many of the offsprings deceased at an age older than 29 years are likely to had children themselves, thus the study participants’ need to combine their own grief with caring for grandchildren and supporting them in their grief may have contributed to the slightly higher AF risk observed in our study participants who lost a child older than 29 years compared with those who lost offsprings of other, non-infant and ages.^7^ The slightly higher point estimate corresponding to the loss of an infant than that corresponding to the loss of other minor children could be attributed to residual confounding by perinatal complications.^2 4^ However, the association did not substantially change after further adjustment for pregestational and gestational hypertension and diabetes, a finding in line with those of several of our earlier studies in this area.^2 4 7^

There are several potential mechanisms by which the death of a child may increase the risk of AF. On the short term, the acute stress following the loss may activate the hypothalamic–pituitary–adrenocortical axis and the autonomic nervous system, which in turn may trigger paroxysmal AF.^9^ Unfortunately, we did not have sufficient statistical power to investigate a triggering effect of bereavement on AF in this study. On the long term, the chronic stress following the death of a child may lead to adverse psychological and behavioural consequences such as depression,^10^ post-traumatic stress disorders^11 12^ and alcohol or drug abuse,^13^ as well as to adverse biological changes including increased inflammation, high heart rate and blood pressure, low heart rate variability,^14–16^ structural and functional changes in the heart, which in turn promote AF.

The strengths of this study include the large sample size, the population-based and prospective design, the long follow-up, and the high-quality information on exposure and outcome, both collected prospectively and independently of each other. However, several limitations should be noted. First, although we adjusted for a large number of covariates and we considered confounding through study design (ie, by performing analyses according to the child’s cause of death), we cannot exclude the possibility of residual confounding. Second, since this study involved only register-based data, we could not investigate whether lifestyle-related and biological mechanisms mediate the association between bereavement and AF. Third, the generalisability of our findings may be limited to countries with a similar sociocultural context and healthcare as that of Denmark. Fourth, though the positive predictive value of the diagnoses of AF in Denmark is high (92.6%), that is, we are likely to have few false positives,^19^ we may have missed some patients with AF, primarily those with asymptomatic and paroxysmal AF, as well as cases diagnosed in specialised outpatient care during 1977–1994. If anything, this misclassification of the outcome is likely to have resulted in an underestimation of the studied association.

## Conclusions

We found that the death of a child was associated with an increased risk of AF. The association was present not only if the child died of CVD but also in case of deaths due to other causes. Further studies are needed to investigate whether our findings may be generalised to other socioeconomic and cultural contexts and to understand the mechanisms underlying the observed association. Bereaved parents may benefit from increased support from family members and health professionals.

## Data Availability

Data are available on reasonable request. All the data used in this study were obtained from Statistics Denmark (https://www.dst.dk/en/kontakt). The data cannot be shared publicly due to the Danish relevant laws and regulations and due to ethical considerations, but it may be requested for research purposes from Statistic Denmark by researchers who fulfill specific requirements.
