# Supplementary material for: Loss of a child and the risk of atrial fibrillation: a Danish population-based prospective cohort study
Source: J Epidemiol Community Health. 2023 Mar 1;77(5):322–7. doi: 10.1136/jech-2022-219695 (PMC10086482; doi:10.1136/jech-2022-219695)
Supplement: Supplementary data [file jech-2022-219695supp001.pdf]

Supplementary materials

Supplementary table 1. The International Classification of Diseases codes used to identify the diagnoses and the causes of death*

Diseases	ICD-8	ICD-10
**Medical condition**		
Atrial fibrillation	427.93, 427.94	I48
Psychiatric disorders	290-315	F00-F99
Hypertension	400-404, 63700, 63703, 63704, 63709, 63719	I10-I15, O10, O11, O13-O16
Diabetes	249, 250	E10-E14, O24
CVD	390-458	I00-I99
**Cause of death**		
Death due to CVD	390-458	I00-I99
Death due to other causes	All other codes	All other codes

CVD=cardiovascular diseases; ICD=International Classification of Diseases.
*Denmark used ICD-8 from 1970 to 1993 and ICD-10 since 1994.

Supplementary table 2. The Danish national registers used to retrieve information for the study

Variables	Source of information	Period covered
**Child's characteristics**		
Date of birth	Medical Birth Register	1973-2016
Maternal smoking in early pregnancy	Medical Birth Register	1991-2016
Maternal hypertension before childbirth	National Hospital Register*	1977-2016
Maternal diabetes before childbirth	National Hospital Register	1977-2016
Date of death	Civil Registration System	1970-2016
Cause of death	Civil Registration System	1970-2016
**Study participants' characteristics**		
Age	Civil Registration System	1970-2016
Sex	Civil Registration System	1970-2016
Country of birth	Civil Registration System	1970-2016
Marital status	Civil Registration System	1972-2016
Linkage to children	Civil Registration System	1970-2016
Linkage to parents and siblings	Civil Registration System	1970-2016
Education	Integrated Database for Longitudinal Labor Market Research	1980-2016
Income	Integrated Database for Longitudinal Labor Market Research	1980-2015
History of CVD	National Hospital Register	1977-2016
History of psychiatric disorders	National Hospital Register<br>Central Psychiatric Register†	1969-2016
Parents' and siblings' history of CVD	National Hospital Register	1977-2016

* The information on inpatient care in the National Hospital Register was available for the period 1977-2016 and that on outpatient care for the period of 1995-2016.
† The information on inpatient care in the Central Psychiatric Register was available for the period 1969-2016 and that on outpatient care for the period of 1995-2016. In addition, all psychiatric inpatient, outpatient and emergency department contacts in Denmark have been reported to the National Hospital Register since 1995.

Supplementary table 3. Adjusted incidence rate ratios and 95% confidence intervals for the association between the death of a child and the risk of atrial fibrillation in different subgroups

Subgroups	Number of events	Rate/10^5 person-year	Multivariable IRR (95% CI)	P-value for the interaction
**Sex**				
Mothers	22,482	72.3	1.14 (1.06-1.22)	Reference
Fathers	52,223	176.7	1.10 (1.04-1.15)	0.0217
**Age***				
<50 years	8137	23.1	1.21 (1.02-1.45)	Reference
≥50 years	66,388	269.7	1.12 (1.07-1.17)	0.4156
**Education at baseline†**				
0-9 years	24,898	130.9	1.18 (1.11-1.25)	Reference
10-14 years	34,300	119.7	1.04 (0.97-1.11)	0.0029
≥15 years	12,777	121.1	1.15 (1.03-1.29)	0.6415
**Study entry categorized at the time when the NHR became nationwide**				
Before 1978	45,311	210.4	1.11 (1.06-1.16)	Reference
1978 and afterwards	29,394	75.2	1.14 (1.06-1.23)	0.5965
**Study entry categorized at the time when the NHR included specialized outpatient care**				
Before 1995	4500	21.4	1.24 (0.99-1.56)	Reference
1995 and afterwards	70,205	177.1	1.12 (1.07-1.17)	0.4273

IRR=incident rate ratio; CI=confidence interval; NHR=National Hospital Register.
* Adjusted for sex, age at follow-up, calendar year at follow-up, country of birth, highest educational attainment, history of psychiatric disorders and of cardiovascular diseases.
† Adjusted for sex, age at follow-up, calendar year at follow-up, country of birth, history of psychiatric disorders and of cardiovascular diseases.

Supplementary table 4. Adjusted incidence rate ratios and 95% confidence intervals for the association between the death of a child and the risk of atrial fibrillation in sensitivity analyses

Study participants	Sensitivity analyses	N	Multivariable IRR (95% CI)
Participants with information on marital status	Main model	1,896,226	1.09 (1.04-1.14)
	Main model + marital status	1,896,226	1.09 (1.04-1.14)
Participants with information on income	Main model	1,887,599	1.15 (1.05-1.26)
	Main model + income	1,887,599	1.15 (1.05-1.25)
Participants who did not lose a child prior to baseline	Main model	2,798,097	1.12 (1.08-1.17)
Participants with linkage to their parents	Main model	1,933,250	1.20 (1.12-1.30)
	Main model + family history of CVD	1,933,250	1.21 (1.12-1.30)
All female study participants	Main model	1,411,594	1.14 (1.06-1.22)
	Main model + pregestational and gestational hypertension and diabetes	1,411,594	1.14 (1.06-1.22)
Mothers with information on maternal smoking in early pregnancy at baseline*	Main model	693,990	1.21 (0.84-1.74)
	Main model + maternal smoking in early pregnancy	693,990	1.21 (0.84-1.73)

IRR=incidence rate ratio; CI=confidence intervals; CVD=cardiovascular diseases.
The main model was adjusted for sex, age at follow-up, calendar year at follow-up, country of birth, highest educational attainment, history of psychiatric disorders and of cardiovascular diseases.
*Information on maternal smoking in early pregnancy was available in the Danish Medical Birth Register since 1991.
